# Growth, productivity and phytochemicals of Coriander in responses to foliar application of *Acacia saligna* fruit extract as a biostimulant under field conditions

**DOI:** 10.1038/s41598-024-53378-5

**Published:** 2024-02-05

**Authors:** A. A. Alkharpotly, Doaa Y. Abd-Elkader, Mohamed Z. M. Salem, Hanaa S. Hassan

**Affiliations:** 1 Horticulture Department, Faculty of Agriculture and Natural Resources, Aswan University, Aswan, Egypt; 2 Horticulture Department, Faculty of Desert and Environmental Agriculture, Matrouh University, Marsa Matrouh, Egypt; 3 Department of Vegetable, Faculty of Agriculture (EL-Shatby), Alexandria University, Alexandria, 21545 Egypt; 4 Forestry and Wood Technology Department, Faculty of Agriculture (El-Shatby), Alexandria University, Alexandria, 21545 Egypt

**Keywords:** Chemical biology, Plant sciences

## Abstract

The application of natural extracts to vegetable plants can increase production, optimize nutrient and water uptake, and mitigate the effects of stress on vegetable plants by enhancing primary and secondary metabolism. In this study, *Acacia saligna* (Labill.) H.L.Wendl. fruit aqueous extract (FAE) was applied as a foliar application to assess and demonstrate its effects on growth, productivity, and phytochemicals of coriander (*Coriandrum sativum* L.) plants. *A. saligna* FAE (2%, 4%, and 6%), each combined with 50% of the recommended dose of N fertilizer was applied to coriander plants over the course of two successive seasons in the field. These treatments were compared with the control treatment, which used a 100% recommended dose of N. The four tested treatments were set up in a randomized complete block design with three replicates for a total of 12 experimental plots. Each replicate (experimental plot) was 3 m^2^ (2 × 1.5 m^2^) in size and included 300 seeds/m^2^. The phytochemicals were examined using chromatographic and spectrophotometric methods, where the essential oils (EOs) extracted from leaves were analyzed by Gas chromatography–mass spectrometry (GC–MS), while the phenolic and flavonoid compounds were analyzed by High Performance Liquid Chromatography (HPLC). With the application of *A. saligna* FAE (4%) + 50% N fertilizer, the levels of total solid content, total carbohydrates, total protein, total phenols, and total antioxidant activity, as well as chlorophyll a, chlorophyll b, chlorophyll a + b, and carotenoids, were increased at harvest. The treatment *A. saligna* FAE at 6% + 50% N fertilizer did not observe significant improvement in the growth parameters of coriander plants because of the anticipated allelopathic effects. By GC–MS analysis, the major compounds in the EO from control treatment were 2-octyn-1-ol (23.93%), and 2-butyl-1-octanol (8.80%), in treated plants with 2% of *A. saligna* FAE + 50% N fertilizer were (*E*)-2-decen-1-ol (32.00%), and 1-methoxymethoxy-oct-2-yne (13.71%), in treated plants with 4% *A. saligna* FAE + 50% N fertilizer were *E*-2-undecen-1-ol (32.70%), and 3,5,5-trimethyl-1-hexene (8.91%), and in the treated plants with *A. saligna* FAE (6%) + 50% N fertilizer were phytol (80.44%), and (*Z*)6,(*Z*)9-pentadecadien-1-ol (13.75%). The flavonoid components 7-hydroxyflavone, naringin, rutin, quercetin, kaempferol, luteolin, apigenin, and catechin were presented with variable concentrations according to the treatments utilized as identified by HPLC analysis from the methanol extracts of the treated plants with the combination treatments of *A. saligna* FAE (2, 4, and 6%) and N fertilization (50% from the recommended dose) and control coriander plants (100% N recommended dose). The combination of 50% N fertilizer treatment and the biostimulant *A. saligna* FAE (4%) seems to improve coriander plant growth while simultaneously lowering N fertilizer consumption. Future research will be needed to further study the effectiveness of several concentrations of *A. saligna* FAE in various conditions and/or species.

## Introduction

As a member of Apiaceae family, *Coriandrum sativum* L. (coriander) is one of the most beneficial spices and medicinal herbs^1^. Hippocrates (460–377 BC) used the essential oil (EO) from coriander in time-honored Greek treatments, and it has a historic medicinal use^2,3^. It is an annual herb that indigenous to the Mediterranean and Western Asia regions. The main nations that produce coriander plants are Syria, Canada, China, India, Morocco, Bulgaria, Egypt, and Romania. More than 80% of the world’s coriander production is produced in India^4^. In Egypt, the average amount of coriander crop production was 9336.8 tons between 1990 and 1997, and 13,094 tons between 1998 and 2004^5,6^ and 3496 ton in 2017^7^. According to data from 2008–2009, coriander is grown on about 8079 feddan, with a production of about 7292 tons^8^, while throughout the years 2000–2016, the coriander crop was grown on an average of roughly 9.50 thousand feddan^7^. The total exported quantity of Egyptian coriander during the period 2009–2011 was 1539 tons^8^, while it was 1181.6 tons during the period 2000–2017^7^.

Due to its widespread use from the Middle East across all of southern Asia and the majority of Latin America, green coriander leaves have been dubbed the most widely used herbal flavoring in the creation of salad and sauces worldwide^9,10^. Cilantro, also known as Chinese parsley, is frequently used in Chinese, South American, Mexican, and Indian cuisines^11^. Due to their high concentration of health-promoting metabolites such as antioxidants, chlorophylls, carotenoids, certain elements, fibers, vitamin C, vitamin A, as well as their low-fat content, green coriander leaves play a significant role in the diet^1^.

One of the constraints restricting modern agriculture is the use of chemical nitrogen, which must be reduced to reduce the negative economic and environmental effects of fertilization as well as the accumulation of nitrate^12–14^. According to studies by Alberici et al.^15^ and Cavaiuolo and Ferrante^16^, increased nitrate availability in leafy vegetables frequently leads to an accumulation in leaves with nitrate levels that are higher than those permitted by EU regulations. High N fertilizer rates can harm the environment by increasing greenhouse gas emissions of nitrous oxide and nitrate flows into streams^14,17,18^. Fruits and vegetables can accumulate high amounts of nitrates, which can have a negative effect on human health^19,20^. Utilizing a biostimulant made from plant extracts collected from various arboreal plant parts to increase nitrogen uptake while maintaining yields^21,22^.

Biostimulants are tools for sustainable agriculture since they are eco-friendly particularly in developing agricultural nations, where the usage of mineral fertilizers places a heavy financial burden on farmers^23^. By definition, biostimulants are “substances or mixtures of particles or microorganisms that, when used with plants, are capable of improving trophic efficiency, abiotic stress tolerance, and/or crop quality traits”^24,25^. As a result, biostimulants are employed as growth promotors and as supplements to mineral fertilizers since they help to increase growth, yield and crop quality^26–30^. By promoting root growth and increasing the antioxidant capacity of plants, biostimulants application in vegetables permitted a decrease in fertilizers and an increase in leaf pigments (chlorophyll and carotenoids) and plant growth^31–33^.

Low dosages of bio-based products, such as plants, seaweed, and agricultural wastes, might be a viable way to reduce fertilizer usage while simultaneously enhancing plant development^34^. In this context, plant extracts are a potential class of biostimulants of vegetal origin that improve vegetable quality in terms of phytochemical content and crop performance by enhancing nutrient availability, absorption, and metabolic utilization^35–37^. The effects of plant extracts are brought about by their interference with the phytohormone balance in plants, according to Paradikovi’c^38^. Additionally, plant extracts are designed to function as defense mechanisms against abiotic stress since they include bioactive molecules that control plant physiology and metabolism. This can help minimize yield losses brought on by unfavorable soil conditions^39,40^. Treatments with soybean leaf extract raised the Mg and Ca concentrations in lettuce, whereas Chinese chive treatments enhanced the Fe contents^41^. Furthermore, Chinese chive, soybean leaf, and soybean stem extracts raised the levels of total and free amino acids in test plants while also increasing the glucose and maltose contents in lettuce plants following treatments^41^.

Numerous treatments and biostimulants have a substantial impact on the vegetative characteristics, oil content, and biochemical components including ascorbic acid, total chlorophyll, and carotenoids of the *C. sativum* and other horticultural plants. The administration of biostimulants to chilled plants caused an increase in L-ascorbic acid, total phenolic content, and overall antioxidant activity^42^. The biostimulants can also alter certain metabolic pathways, speeding up the adaption of chilled coriander plants^42^. With 4% of moringa leaves extract (MLE), pea plants (*Pisum sativum* L.) accumulated the most nutrients, growth indices, and photosynthetic pigment^43^. By enhancing leaf pigments and photosynthetic activity as well as plant fresh weight after treatments with 10 mL/L dosages of borage extracts, lettuce's primary metabolism was improved^44^.

The matured leaves of coriander contain high levels of moisture, protein, and total ash (1.7%, 87.9%, and 3.3%, respectively), as well as carbohydrates (6.5% total sugar)^45^. The *trans*-tridecen in the EO from immature fruits and leaves of coriander is what gives off the unpleasant odor known as the “stink bug smell”^46^. (E)-2-decenal, is the most prevalent compound in the EOs extracted from the leaves of *C. sativum*, with a concentration of 52%, varied depending on the amount of treatment^47^. 44 chemicals were found in the EO of *C.* *sativum* leaves, the majority of which were aromatic acids such 2-decenoic acid, E-11-tetradecenoic acid, capric acid, undecyl alcohol, tridecanoic acid and undecanoic acid^48^. The seed extract was shown to include phenolic compounds such as gallic acid, caffeic acid, ellagic acid, quercetin, and kaempferol^49^.

Natural extracts from forestry trees have been proven to increase crop and product quality attributes (pre- and post-harvest) nutrient usage efficiency, seed germination, horticultural plant growth and yield and resistance to abiotic stresses^50–52^. Wattle or *Acacia saligna* (Labill.) Wendl, is a woody plant found in the warm climates of the world, including Australia, the Americas, Africa and Asia^53^. It is also described as a woody tree that grows quickly, is invasive, and has a sizable quantity of protein (18.25–35.5%)^53^. *A. saligna* extracts from the stem and leaves contain allelopathic compounds that are thought to be phytotoxic and inhibits plant development or germination^54–56^.

Therefore, the objective of this research was to evaluate the biostimulant effects of *Acacia saligna* fruit aqueous extract as a foliar application in combination with N fertilizer on the vegetative and biochemical parameters, and the essential oil composition of coriander plants.

## Materials and methods

### Experimental conditions and Calypso cultivar properties

At the experimental farm of the Faculty of Agriculture and Natural Resources, Aswan University, Aswan, Egypt, two field experiments on *Coriandrum sativum* L. were carried out in the winter of 2020 and 2021. The site is located at 23° 59′ 56″ N, 32° 51′ 36″ E, with an average elevation of 85 m above sea level. The average temperatures; High 25–30 °C, and Low 12–14 °C, the relative humidity (RH 40%), the wind speed in winter season blows at an average speed of 13.5 mph (21.8 kph) and average wind speed of 13.0 mph (20.9 kph).

The Calypso cultivar, which was obtained from Harraz Company vegetable seed (Cairo, Egypt), was used in this study. It may grow huge, uniform leaves of a consistent color, endure high temperatures, moderate bolting, and have around 110 seeds/g. This cultivar was recommended in some works^57,58^.

### Soil analysis of the experimental site

Following the prescribed protocols, soil samples were taken from the experimental planting site in both seasons at depth of 30 cm and examined for physical and chemical characteristics^59^. Table 1 lists the major physical and chemical soil parameters for the two trial sites.

**Table 1:** Soil analysis of the experimental site during both seasons.

Parameter	Experimental season	
	2020	2021
Soil type	Sandy silt	Sandy silt
pH	8.15	8.25
EC (dSm^−1^)	1.52	2.00
Total N (%)	1.02	1.3
Available P (ppm)	7.50	7.80
Available K (ppm)	170	175

### Preparation of *Acacia saligna* (Labill.) H.L.Wendl. fruit aqueous extract

*A. saligna* (Labill.) H.L.Wendl. trees grown in Abies Station Farm, Alexandria, Egypt (31°12N 29°55E), were chosen and identified at the Department of Forestry and Wood Technology. *A. saligna* fruits (legumes with seeds) were taken after they were fully developed, air-dried in a lab conditions for 4 weeks, and then ground into a fine powder using a small laboratory mill. Approximately 100 g of the ground fruits were put in a conical flask containing 300 mL of distilled water (DW), then extracted for 3 h under heating using a water bath at 50 °C^60^. The extract was concentrated to a small volume after being run through cotton plugs, and filtered by Whatman No. 1 filter paper. The fruit aqueous extract (FAE) was prepared at the concentration of 2%, 4%, and 6%, by dissolving the respective amount in DW.

### Experimental procedure and treatments

In both seasons, coriander seeds were sown from the beginning of November to the end of December. The prepared concentrations (2, 4 and 6%) of *A. saligna* FAE were evaluated in combination with nitrogen fertilizer rate (50% from recommended dose) to measure their effects on growth, productivity, and phytochemicals of coriander. At 20, 30, and 40 days following sowing, the plants were sprayed with foliar treatments (2, 4 and 6% of *A. saligna* FAE) three times. Each experiment included four treatments as shown in Table 2. In a randomized complete block design, the four tested treatments were set up with three replicates, totaling 12 experimental plots. Each replicate (experimental plot) had a surface area of 3 m^2^ (2 × 1.5 m^2^) and included 300 seeds/m^2^. After 20 and 40 days of sowing, the source of N fertilizer, (NH_4_)_2_ SO_4_ (20.5% N), was added twice to the soil.

**Table 2:** Combination treatments of *A. saligna* extract and N fertilizer used for coriander plants.

	Combination treatments
T_1_	Control (100% recommended dose of nitrogen = 240 kg N/ha)
T_2_	*A. saligna* FAE at 2% concentration + 50% (recommended dose 120 kg N/ ha)
T_3_	*A. saligna* FAE at 4% concentration + 50% (recommended dose 120 kg N/ ha)
T_4_	*A. saligna* FAE at 2% concentration + 50% (recommended dose 120 kg N/ ha)

### Vegetative and biochemical parameters

#### Vegetative parameters

To assess the vegetative development, 10 plants were randomly selected from each experimental unit at harvest time in both seasons to measure plant height (cm), number of leaves/plant, plant fresh weight (g) and total yield (kg/m^2^).

#### Measuring of photosynthetic pigments

The extraction of chlorophylls (a, b and a + b) and carotenoids, which are photosynthetic pigments, were extracted from leaves using ethanol 96% (v/v) in a ratio of 1:10 (w/v). The absorbance was measured at 664 and 649 nm for chlorophylls a and b, respectively, and 470 nm for carotenoids^61^.

#### Measuring of total soluble solids, total carbohydrates and crude protein content

A portable digital refractometer (Atago Co. Ltd., modelPR-1, Tokyo, Japan) was used to measure the total soluble solids (TSS %) in the leaf juice. The percentage of total carbohydrates (%) in leaves' dry matter was calculated^62^. The approved Kjeldahl technique outlined in AOAC^62^, was used to calculate the crude protein content.

#### Total phenolic content and antioxidant activity

The total phenolic content (TPC) was determined by Folin–Ciocalteu reagent and the absorbance was measured by Optizin UV–Vis spectrophotometer model (Thermo Electron Corporation, Waltham, MA, USA) at 750 nm^63^. Using the gallic acid calibration curve, the absorbance was converted to gallic acid equivalent (GAE). The antioxidant activity was evaluated using the 2,2-diphenyl-1-picrylhydrazyl (DPPH) assay^64^.

### Extraction of essential oils and Gas chromatography–mass spectrometry (GC–MS) analysis

The essential oils (EOs) from leaves of coriander plants treated with the four treatments were extracted using a Clevenger-type apparatus through the hydrodistillation method^65^. To do this, 1 L of distilled water was added to a 2 L flask containing 100 g of leaves and then connected to Clevenger-type apparatus with the condenser and heated continuously for 3 h at a temperature of 120 °C. We proceeded with the extraction of the EOs using the method of Shahwar et al.^66^ approach because it has already been mentioned that the volatiles from coriander leaves was extremely low.

#### Analysis the essential oils by gas chromatography–mass spectrometry (GC–MS)

Thermo Scientific's Trace GC Ultra-ISQ mass spectrometer, which has a direct capillary column TG-5MS (30 m × 0.25 mm × 0.25 m film thickness), was used to analyze the chemical composition of the EOs. The temperature of the column oven was first maintained at 70 °C, then increased by 5 °C/min to 280 °C, kept for 5 min, and then increased to 300 °C at 5 °C/min. Temperatures were maintained at 250 °C for the MS transfer line and injector. As a carrier gas, helium was employed at a constant flow rate of 1 mL/min. Using an Autosampler AS1310 combined with GC in split mode, diluted samples of 1 L were automatically injected with a solvent delay of 2 min. Full scan EI mass spectra covering the m/z range of 40–600 were collected at 70 eV ionization voltages. The temperature of ion source was set at 200 °C^67^. By comparing the retention times and mass spectra of the components with those of the WILEY 09 and NIST 11 mass spectral databases and calculating the match factor, the components were identified^68,69^.

#### Chemosystematic significance via multivariate analysis

The chemosystematic significance of this plant was established via the multivariate assessing, comprising principal component analysis (PCA), of the EO principal components of the various *C.* *sativum* plants. The major compounds of the *C.* *sativum* EO (> 2.5%) and those of the other *C.* *sativum* plants around the world were used to construct the chemo-systematic significance of the species.

### Leaf methanol extracts

For the extraction, leaf samples from the four treated *C.* *sativum* plants (Table 2) were obtained. Each of the treated leaves weighed about 50 g, and 150 mL of methanol was used to extract them over the course of a week at room temperature by the soaking method^26^. The extracts were then filtered using a cotton plug and Whatman no. 1 filter paper. Prior to usage, the extracts were concentrated and kept in the refrigerator in brown vials.

### Analysis of flavonoid and phenolic compounds by HPLC

With the use of High Performance Liquid Chromatography (HPLC) system (Agilent 1100), which consists of two LC pumps, a UV/Vis detector, and a C18 column (250 mm × 4.6 mm, 5 µm), flavonoid components from the methanol extracts of the four treated *C.* *sativum* plants were identified. With an isocratic elution (70:30) program, the mobile phase consisted of acetonitrile (A) and 0.2% (v/v) aqueous formic acid (B). The detection wavelength was set at360 nm^70^.

Using HPLC, The phenolic and flavonoid components from *Acacia saligna* FAE were identified by HPLC analysis^70^. For instance, to analyze the phenolic compound, HPLC (Agilent 1100, Agilent ChemStation) had a UV/Vis detector, two LC pumps, and a C18 column (125 mm × 4.6 mm, 5 µm particle size) was used to gather and examine chromatograms. By using a gradient mobile phase of two solvents—Solvent A (Methanol) and Solvent B [Acetic acid in water (1:25)], phenolic compounds were isolated. For the first 3 min, the gradient program was maintained at a concentration of 100% B. The concentration of eluent A was then raised to 80% for the following 2 min, then decreased to 50% once again for the following 5 min detection wavelength at 250 nm. This was followed by 5 min of 50% eluent A. As a result, the order of phenolic compounds was established utilizing this mobile phase to verify standard compounds.

### Statistical analysis

All the collected data were analyzed using the CoStat software version 6.303^71^ and the treatments means were compared using the LSD test at 0.05 level of probability^72^.

### Ethics approval and consent to participate

This study is complied with relevant institutional, national, and international guidelines and legislation. This study does not contain any studies with human participants or animals performed by any of the authors.

## Results

### Measurement of vegetative growth and total yield parameters

**Figure 1:**
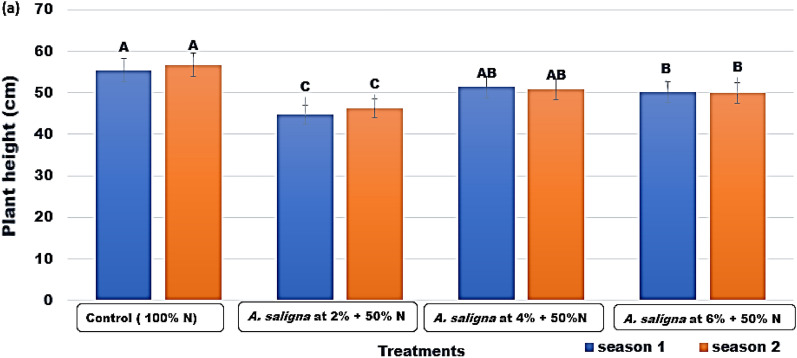
Plant height (cm) of *C.* *sativum* plants treated with *A. saligna* FAE + N fertilizer. Letters in Figure indicated that, means ± S.E of treatments with the same letter/s were not significantly different according to LSD at 0.05 level of probability. LSD 0.05 for Season 1 = 2.38 and Season 2 = 1.98.

The plants treated with the control treatment (Fig. 1a) had the maximum plant height values of 55.4 and 56.7 cm in both seasons, followed by the plants treated with 4% and 6% of *A. saligna* FAE + 50% N fertilizer. Plants treated with *A. saligna* FAE 2% + 50% N fertilizer treatment had the lowest plant height values..

**Figure 2:**
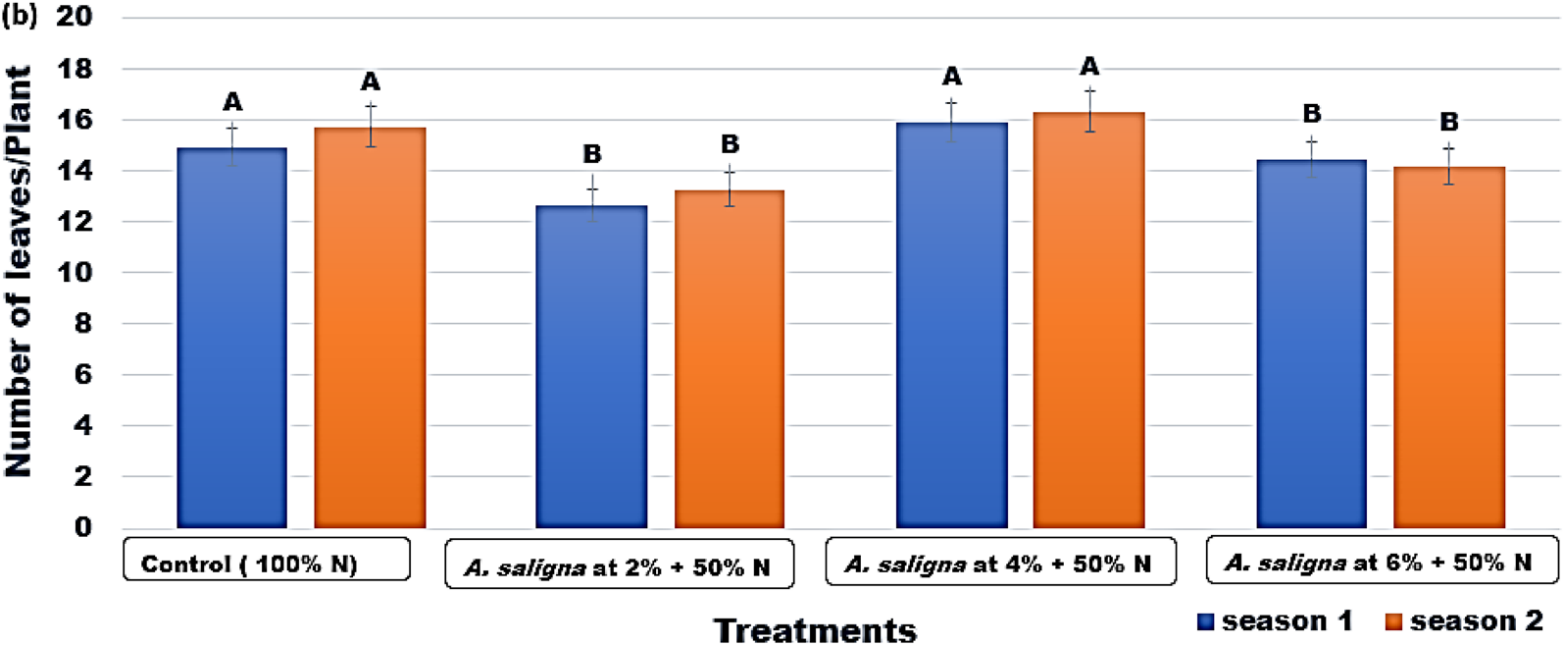
The number of leaves/plant of *C.* *sativum* plants treated with *A. saligna* FAE + N fertilizer. Letters in Figure indicated that, means ± S.E of treatments with the same letter/s were not significantly different according to LSD at 0.05 level of probability. LSD 0.05 for Season 1 = 1.01 and Season 2 = 1.50.

The maximum number of leaves were produced by the plants treated with *A. saligna* FAE 4% + 50% N fertilizer with values of 15.9 and 16.3 leaf/plant in both seasons, respectively (Fig. 2).

**Figure 3:**
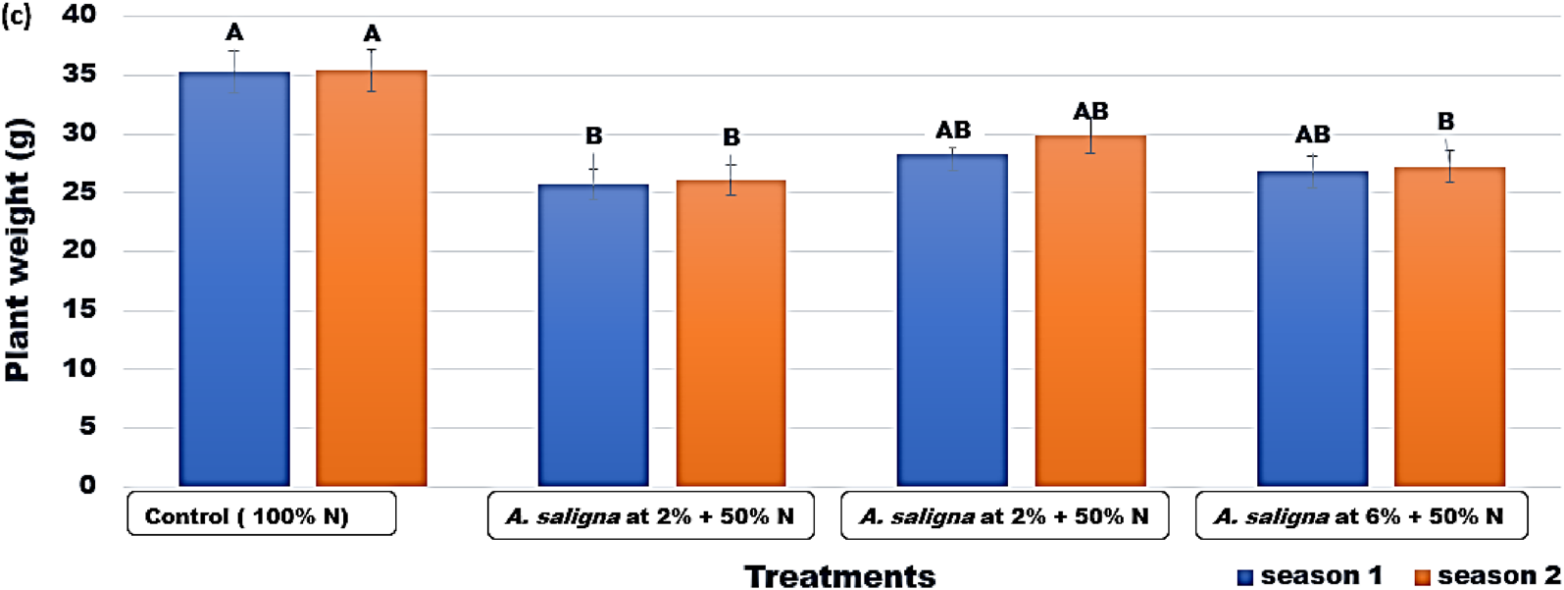
The plant weight (g) of *C.* *sativum* plants treated with *A. saligna* FAE + N fertilizer. Letters in Figure indicated that, means ± S.E of treatments with the same letter/s were not significantly different according to LSD at 0.05 level of probability. LSD 0.05 for Season 1 = 5.20 and Season 2 = 5.71.

**Figure 4:**
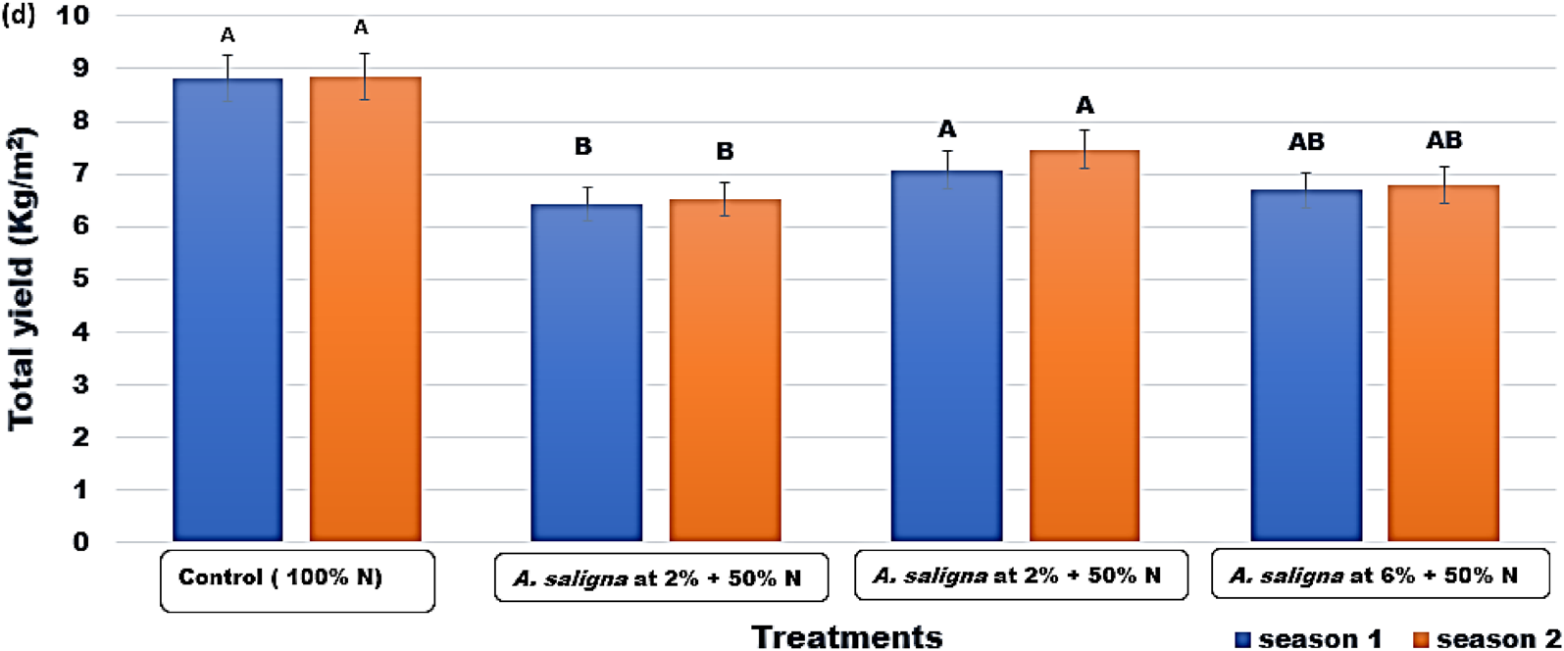
Total yield (kg/m^2^) of *C.* *sativum* plants treated with *A. saligna* FAE + N fertilizer. Letters in Figure indicated that, means ± S.E of treatments with the same letter/s were not significantly different according to LSD at 0.05 level of probability. LSD 0.05 for Season 1 = 1.07 and Season 2 = 1.39.

The control plants had the maximum plant weight in both seasons, measuring 35.30 and 35.44 g (Fig. 3), followed by the plants given *A. saligna* FAE 4% + 50% N fertilizer in each season. While plants treated with *A. saligna* FAE 2% + 50% N fertilizer had the lowest values. The highest total yield was observed in the control treatment with values of 8.82 and 8.88 kg/m^2^, in both seasons, respectively (Fig. 4).

### Photosynthetic pigments measurements and biochemical compounds

**Figure 5:**
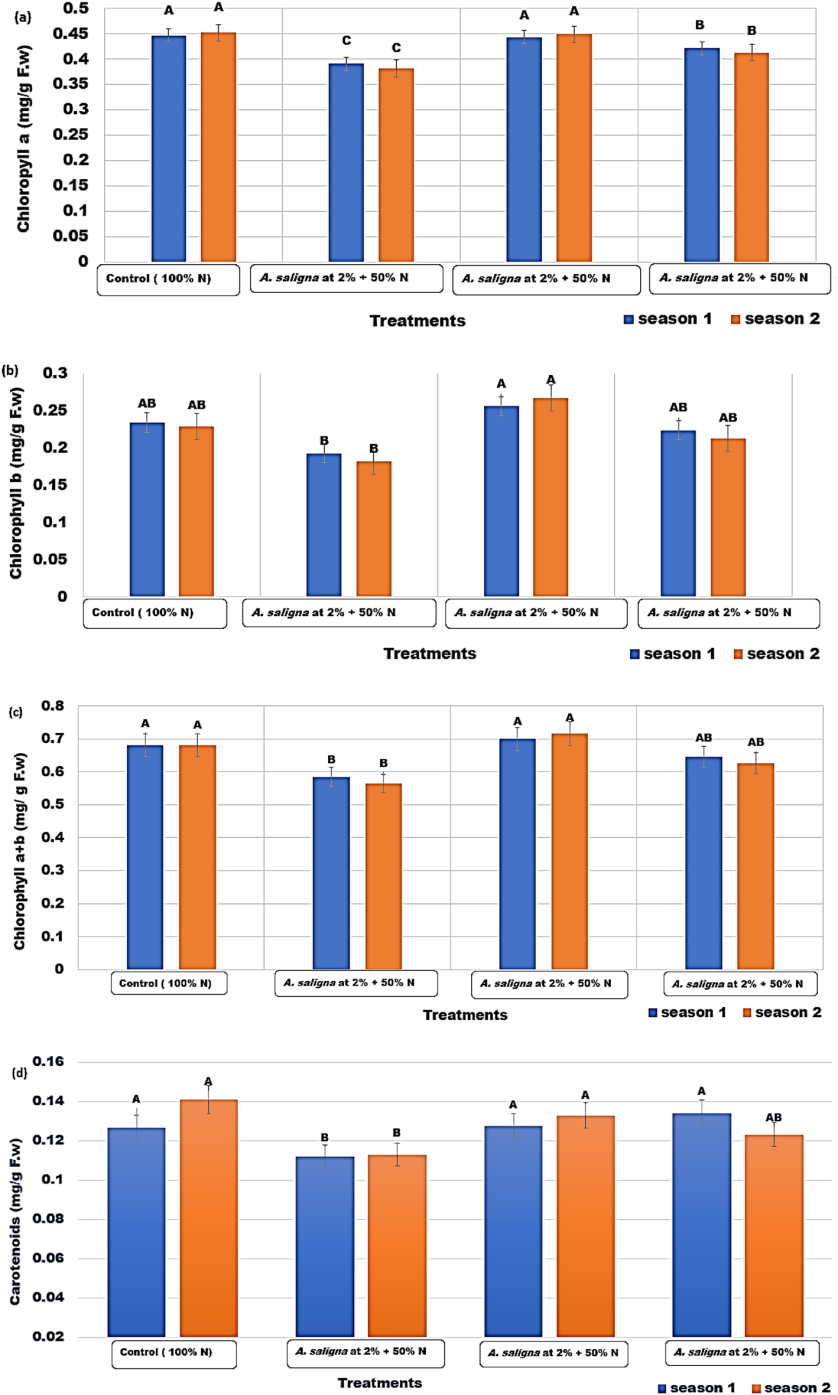
Characterization of photosynthetic pigments of *C.* *sativum* plants as affected by the treatments of *A. saligna* FAE + N fertilizer. (**a**) Chlorophyll a content (mg/g fw); (**b**) Chlorophyll b content (mg/g fw); (**c**) Chlorophylls a + b (mg/g fw), and (**d**) Carotenoids (mg/g fw). Letters in Figure indicated that, means ± S.E of treatments with the same letter/s were not significantly different according to LSD at 0.05 level of probability.

Chlorophyll a, chlorophyll b, and chlorophylls a + b contents in the leaves of coriander plants treated with *A. saligna* FAE (2, 4, and 6%) + 50% N fertilizer are shown in Fig. 5a–c, respectively, along with a comparison to the control plants (treated with 100% N) in both seasons. The highest content of Chlorophyll a was found with the treated plants with *A. saligna* FAE 4% + 50% N fertilizer (Fig. 5a) in both seasons with values of 0.443 and 0.449 mg/g fw, respectively. Chlorophyll b content (Fig. 5b) reached the highest content in both seasons as the plants treated with *A. saligna* FAE 4% + 50% N fertilizer with values of 0.256, and 0.267 mg/g fw, respectively. The highest values of chlorophyll a + b content was obtained in plants treated with 4% *A. saligna* FAE + 50% N fertilizer, in both seasons, which reached 0.699 and 0.716 mg/g fw, respectively (Fig. 5c).

The highest level of carotenoids (Fig. 5d) in the first season was observed in plants treated with *A. saligna* FAE 6% + 50% N fertilizer (0.134 mg/g fw), which was not statistically significant from those plants treated with *A. saligna* FAE 4% + 50% N fertilizer (0.128 mg/g fw) and in control plants (0.127 mg/g fw). In the second season, the highest values were found in control plants (0.141 mg/g fw), followed by the treated plants with *A. saligna* FAE 4% + 50% N fertilizer (0.133 mg/g fw).

**Table:** 

LSD 0.05	Chlorophyll a content (mg/g fw)	Chlorophyll b content (mg/g fw)	Chlorophylls a + b (mg/g fw)	Carotenoids (mg/g fw)
Season 1	0.021	0.051	0.086	0.011
Season 2	0.027	0.072	0.092	0.012

### Measurements of biochemical compounds, total phenolic and antioxidant activity

The total soluble solids (TSS), total carbohydrates, total protein, total phenolic compounds (TPCs), and the total antioxidant activity (TAA) of coriander leaf are shown in the Table 3. *A. saligna* FAE (4%) + 50% N fertilizer-treated plants had the highest TSS levels in both seasons, with values of 8.8 and 8.9%, respectively.

**Table 3:** Biochemical compounds, the total phenolic content and antioxidant activity of coriander leaf.

Treatment	TSS (%)		Total carbohydrates (%)		Total protein (%)		TPC (mg GAE/100 g fw)		TAA (%)	
	Season 1	Season 2	Season 1	Season 2	Season 1	Season 2	Season 1	Season 2	Season 1	Season 2
T1	7.3 ± 0.2AB	7.5 ± 0.2AB	22.30 ± 0.1A	23.51 ± 0.4A	15.92 ± 0.03A	15.31 ± 0.02A	261.46 ± 0.01A	260.23 ± 0.05A	69.49 ± 0.1AB	66.45 ± 0.22B
T2	5.9 ± 0.1B	5.4 ± 0.2B	20.22 ± 0.5B	19.91 ± 0.3AB	10.02 ± 0.01B	11.23 ± 0.01B	210.01 ± 0.03B	212.66 ± 0.05B	50.42 ± 0.2C	52.33 ± 0.31C
T3	8.8 ± 0.3A	8.9 ± 0.2A	24.36 ± 0.6 A	25.21 ± 0.2A	16.32 ± 0.01A	16.16 ± 0.03A	266.54 ± 0.04A	269.22 ± 0.03A	78.30 ± 0.5A	79.53 ± 0.5A
T4	7.8 ± 0.2AB	7.7 ± 0.1AB	21.14 ± 0.1AB	22.12 ± 0.1A	15.22 ± 0.02A	15.01 ± 0.01A	248.86 ± 0.01AB	236.26 ± 0.02AB	60.11 ± 0.02B	61.22 ± 0.11B
LSD 0.05	2.17	2.52	3.36	4.14	2.11	2.23	53.28	45.72	10.25	11.31

Values are mean ± SE. Means with the same letter/s within the same column are not significantly different according to LSD at 0.05 level of probability.

*TSS* Total soluble content, *TAA* Total antioxidant activity, *TPC* Total phenolic content, *GAE* Gallic acid equivalent, *T1* Control, *T2 A. saligna* FAE 2% + 50% N fertilizer, *T3 A. saligna* FAE 4% + 50% N fertilizer, *T4 A. saligna* FAE 6% + 50% N fertilizer.

The total carbohydrates was presented in the highest amount in plants treated with of *A. saligna* FAE 4% + 50% N fertilizer, with percentages of 24.36% and 25.21% in each season, respectively (Table 3).

The highest content of protein was found in leaves of plants treated with *A. saligna* FAE 4% + 50% N fertilizer with values of 16.32% and 16.16%, respectively, in both seasons (Table 3).

The plants treated with *A. saligna* FAE 4% + 50% N fertilizer had the highest total phenolic content with values of 266.54, and 269.22 mg GAE/100 g fw, respectively, in both seasons (Table 3).

The leaf methanol extract from plants treated with *A. saligna* FAE 4% + 50% N fertilizer had had the highest total antioxidant activity as determined by the DPPH method with values of 78.30, and 79.53%, in both seasons, respectively (Table 3).

### Leaf essential oil compositions

The percentages of the obtained essential oils (EOs) were in the samples collected from untreated, and In the samples taken from treated plants with 2%, 4%, and 6% *A. saligna* FAE + 50% N fertilizer, respectively, and control plants, the percentages of the obtained EOs were 0.06, 0.13%, 0.09% and 0.08%. Table 4 displays the chemical composition of the EOs derived from *C.* *sativum* leaves as influenced by various treatments of *A. saligna* FAE + 50% N fertilizer compared the control plants that treated with 100% recommended dose of N (240 kg N/ha).

**Table 4:** Chemical compounds of the essential oils from *C. sativum* leaves as affected by various treatment.

Compound	Compound percentage (%) in the EO from plants treated with			
	Control (100% N)	2% of *A. saligna* FAE + 50% N fertilizer	4% of *A. saligna* FAE + 50% N fertilizer	6% of *A. saligna* FAE + 50% N fertilizer
4-Methyl-2-propylpentan-1-ol	2.66 (829)*	ND	ND	ND
3,5,5-Trimethyl-1-hexene	5.23 (704)	ND	8.91 (877)	ND
Octanoyl chloride	ND	0.56 (673)	ND	ND
2-Nitro-2-hepten-1-ol	ND	0.36 (756)	ND	ND
10-Methyl-E-11-tridecen-1-ol propionate	ND	5.14 (682)	ND	ND
1,2-Epoxynonane	1.80 (757)	0.30 (792)	1.79 (772)	ND
2-Butyl-1-octanol	8.80 (729)	ND	8.85 (793)	1.50 (722)
1-Decanol	1.72 (737)	ND	1.98 (748)	ND
1-Nonanol	2.32 (718)	8.26 (658)	4.12 (728)	ND
(*Z*)-5-Tridecene	2.34 (747)	ND	2.44 (758)	ND
(*E*)-2-Decen-1-ol	1.29 (724)	ND	1.47 (796)	ND
2,4,6,8-Tetramethyl-1-undecene	3.11 (776)	ND	1.31 (912)	ND
1,10-Decane-1,1,10,10-D4-diol	6.07 (750)	ND	4.61 (728)	6.07 (710)
(*E*)-4-Dodecene	0.65 (796)	ND	0.67 (763)	ND
1-Decene	2.68 (743)	1.13 (736)	2.83 (741)	ND
5,7-Dodecadiyn-1,12-diol	1.59 (763)	2.72 (677)	ND	ND
9-Oxabicyclo[6.1.0]nonan-4-ol	ND	ND	1.79 (715)	ND
2-Nitro-(*Z*)-1,4-nonadiene	ND	0.61 (656)	ND	ND
2-Dodecenal	7.67 (705)	ND	ND	ND
8-Azabicyclo[5.1.0]octane	5.56 (747)	ND	ND	ND
*Z*-10-Pentadecen-1-ol	ND	ND	1.57 (740)	ND
Dodecyl-oxirane	5.32 (746)	ND	4.86 (795)	ND
*trans*-2-Undecen-1-ol	1.04 (756)	ND	1.25 (760)	ND
*trans*-2,3-Epoxydecane	1.84 (750)	ND	2.58 (786)	ND
9,12-Octadecadienal	ND	3.74 (739)	4.58 (769)	0.92 (755)
1,2-Cyclooctanediol	1.74 (718)	ND	ND	ND
9-Octadecenal	1.12 (749)	0.66 (664)	1.47 (793)	ND
1-(Ethenyloxy)-octadecane	2.63 (668)	3.83 (735)	2.00 (762)	ND
3-Nonenoic-3,4-d2 acid, methyl ester	ND	ND	ND	1.30 (725)
1-Dodecene	0.93 (710)	ND	1.17 (706)	ND
(*Z*)6,(*Z*)9-Pentadecadien-1-ol	ND	ND	ND	13.75 (769)
1,2-Epoxy-5,9-cyclododecadiene	2.17 (730)	ND	ND	ND
1,12-Tridecadiene	ND	11.21 (686)	ND	ND
2-Octyn-1-ol	23.93 (736)	ND	ND	ND
(*E*)-2-Decen-1-ol	ND	32.00 (761)	ND	ND
*E*-2-Undecen-1-ol	ND	ND	32.70 (676)	ND
Phytol	ND	ND	ND	80.44 (699)
*Trans*-2-Undecenoic acid	ND	11.38 (669)	ND	ND
Methyl 12,13-tetradecadienoate	ND	1.98 (687)	ND	ND
1-Methoxymethoxy-oct-2-yne	ND	13.71 (730)	ND	ND
Methyl 10,12-pentacosadiynoate	3.74 (690)	0.46 (696)	2.81 (681)	1.00 (789)
2-Methyl-10-undecenal	1.22 (722)	ND	1.50 (658)	ND

*ND* Not detected.

*Values are compound percentage (match factor).

In the control treatment, 26 compounds were identified in the EO from leaves, where the highest percentage components were 2-octyn-1-ol (23.93%), 2-butyl-1-octanol (8.80%), 2-dodecenal (7.67%), 8-azabicyclo[5.1.0]octane (5.56%), dodecyl-oxirane (5.32%), 3,5,5-trimethyl-1-hexene (5.23%), methyl 10,12-pentacosadiynoate (3.74%), and 2,4,6,8-tetramethyl-1-undecene (3.11%), while the minor compounds were 1-decene (2.68%), 4-methyl-2-propylpentan-1-ol (2.66%), 1-(ethenyloxy)-octadecane (2.63%), (Z)-5-tridecene (2.34%), 1-nonanol (2.32%), and 1,2-epoxy-5,9-cyclododecadiene (2.17%).

The EO from leaves of *C. sativum* plants treated with 2% *A. saligna* FAE + 50% N fertilizer showed the 17 compounds, were (E)-2-decen-1-ol (32.00%), 1-methoxymethoxy-oct-2-yne (13.71%), trans-2-undecenoic acid (11.38%), 1,12-tridecadiene (11.21%), 1-nonanol (8.26%), 10-methyl-E-11-tridecen-1-ol propionate (5.14%), 1-(ethenyloxy)-octadecane (3.83%), 9,12-octadecadienal (3.74%), and 5,7-dodecadiyn-1,12-diol (2.72%) were presented as main compounds, while methyl 12,13-tetradecadienoate (1.98%), and 1-decene (1.13%) were found with minor percentages.

The treated plants with 4% *A. saligna* extract + 50% N fertilizer showed the presence of 23 compounds, where the highest percentage of compounds were E-2-undecen-1-ol (32.70%), 3,5,5-trimethyl-1-hexene (8.91%), 2-butyl-1-octanol (8.85%), dodecyl-oxirane (4.86%), 1,10-decane-1,1,10,10-D4-diol (4.61%), 9,12-octadecadienal (4.58%), 1-nonanol (4.12%), 1-decene (2.83%), methyl 10,12-pentacosadiynoate (2.81%), trans-2,3-epoxydecane (2.58%), (Z)-5-tridecene (2.44%), and 1-(ethenyloxy)-octadecane (2.00%).

Using the *A. saligna* extract at 6% + 50% N fertilizer, 7 compounds were identified in the EO from leaves of *C. sativum*, where phytol, the most abundant compound in the EO reached 80.44%, followed by other compounds (Z)6,(Z)9-pentadecadien-1-ol (13.75%), and 1,10-decane-1,1,10,10-D4-diol (6.07%).

**Figure 6:**
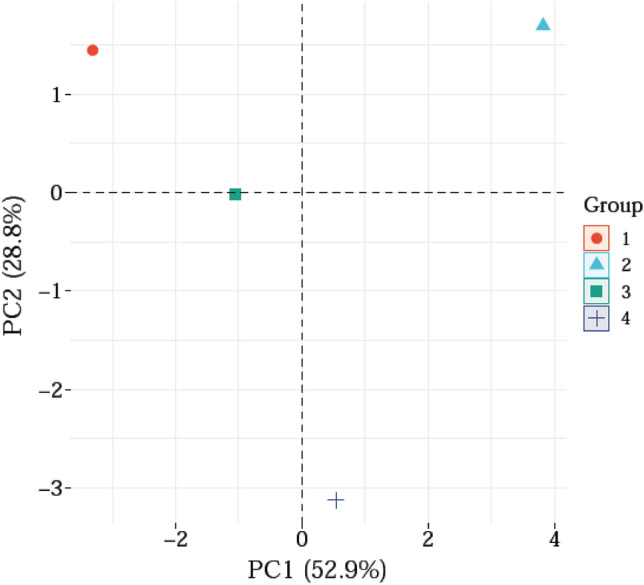
PCA space observation of main constituents of coriander EOs from the 4 treatments.

The foundation of this work was the multivariate analysis of the principal component analysis (PCA). The PCA space observation of main constituents of coriander EOs from the 4 treatments showed that moderate correlation or association ((+ 28.8%) among the EOs identified from the plants extracted from the four treatments (*T1* Control (100% N), *T2* 2% of *A. saligna* FAE + 50% N fertilizer, *T3* 4% of *A. saligna* FAE + 50% N fertilizer and *T4* 6% of *A. saligna* FAE + 50% N fertilizer) (Fig. 6).

**Figure 7:**
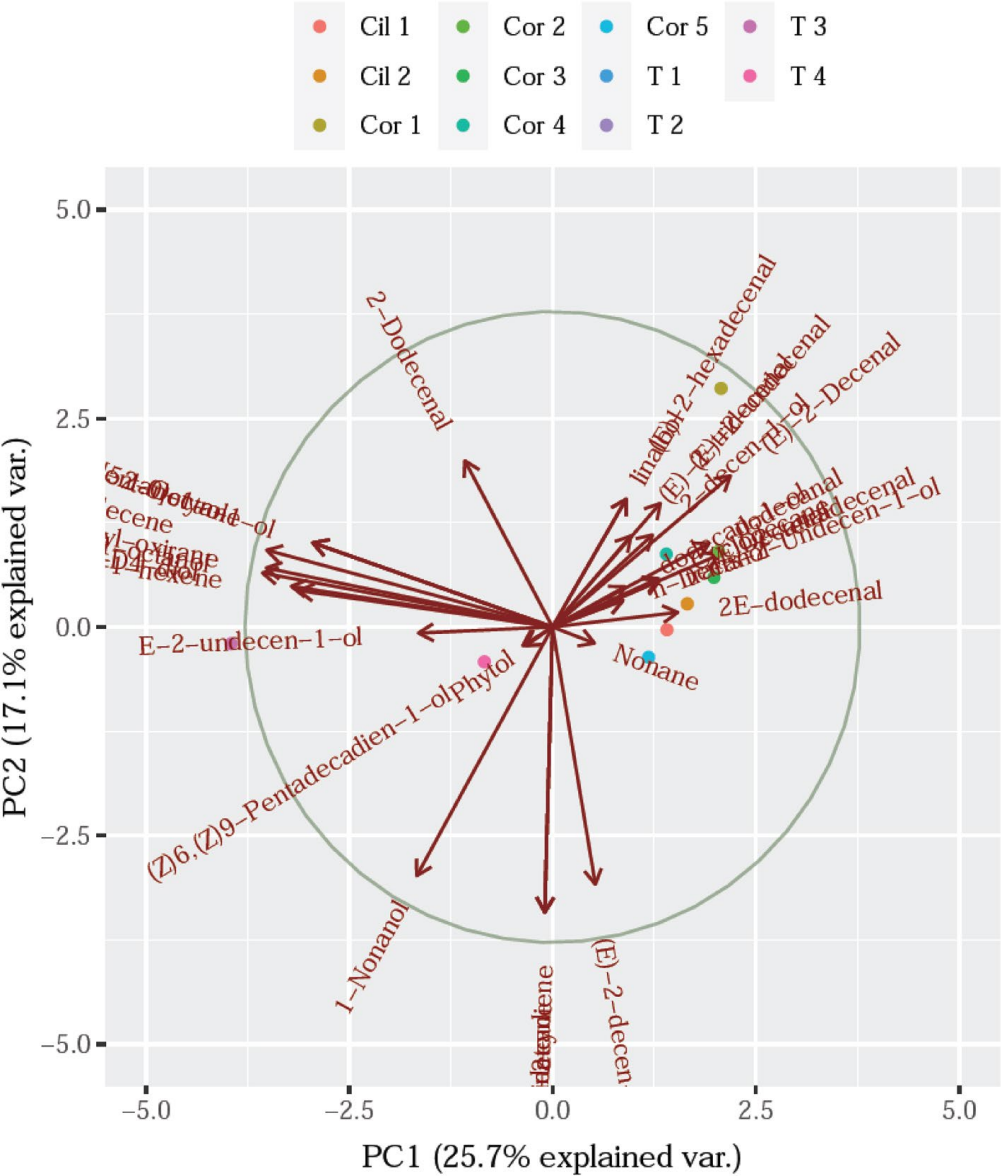
The correlation circle PCA of main constituents of EO of *C*. *sativum* as affected by four treatments and the coriander species including from worldwide.

The correlation circle PCA of main constituents of *C. sativum* EOs as affected by the four treatments was compared with the mains compounds of the EOs from coriander species including from worldwide of the various coriander plants as from Pakistan^66^ (Cor 1), Brazil^73^ (Cor 2) Iran^74^ (Cor 3), Kenya^75^ (Cor 4), Poland^76^ (Cor 5) and two EOs (Cil 1 and Cil 2) from *C*. *sativum* grown from dried fruits (Johnny’s Selected Seeds, Albion, ME)^77^ is shown in Fig. 7.

The PCA analysis showed that *C*. *sativum* has a weak association (+ 17.1%) with the other coriander species. The findings demonstrated that the coriander EOs have a distinctive phenomenon of the existence of 2-Octyn-1-ol (T1), (E)-2-decen-1-ol (T2), E-2-undecen-1-ol (T3) and phytol (T4) as the primary components.

### HPLC analysis of flavonoid components in *C. sativum* leaves

The identified flavonoid components from methanol extracts of *C. sativum* leaves are shown in Table 5 and Fig. S1. In the methanol extract from the control treatment, the two main flavonoid components were catechin (8.65 µg/mL) and luteolin (8.14 µg/mL). The primary flavonoid components present in the methanol extract of leaves from plants treated with 2% *A. saligna* FAE + 50% N fertilizer included luteolin (8.66 µg/mL), naringin (6.54 µg/mL), and kaempferol (5.44 µg/mL). The most prevalent flavonoid component found in the methanol extract of the plants treated with 4% *A. saligna* FAE + 50% N fertilizer was apigenin (9.56 µg/mL). The predominant flavonoid components in the methanol extract of leaves from plants treated with 6% *A. saligna* FAE + 50% N fertilizer were catechin (14.87 µg/mL), luteolin (12.66 µg/mL), and rutin (10.49 µg/mL).

**Table 5:** Flavonoid compounds identified in the leaf methanol extracts of *C. sativum* by HPLC.

Compound	Concentration (µg/mL of methanol leaf extracts) from plants treated with			
	Control	2% *A. saligna* FAE + 50% N fertilizer	4% *A. saligna* FAE + 50% N fertilizer	6% *A. saligna* FAE + 50% N fertilizer
7-Hydroxyflavone	ND	2.33	ND	ND
Naringin	ND	6.54	2.11	ND
Rutin	ND	1.56	ND	10.49
Quercetin	5.66	2.14	3.41	3.20
Kaempferol	ND	5.44	1.45	2.11
Luteolin	8.14	8.66	2.88	12.66
Apigenin	7.02	0.89	9.56	4.09
Catechin	8.65	ND	2.14	14.87

*ND* Not detected.

### Analysis of phenolic and flavonoid compounds from *A. saligna* FAE by HPLC

The identified phenolic and flavonoid constituents in *A. saligna* FAE are shown in Table 6 and Fig. S2. Syringic acid (13.12 μg/g FAE), pyrogallol (9.42 μg/g FAE), and ferulic acid (8.89 μg/g FAE) were the abundant phenolic compounds (Fig. S2a), and apigenin (15.22 μg/g FAE), naringin (12.45 μg/g FAE) and rutin (9.02 μg/g FAE) were the abundant flavonoid compounds (Fig. S2b).

**Table 6:** Phenolic and flavonoid compounds identified fruit aqueous extract from *Acacia saligna* by HPLC.

Phenolic compound	Concentration (μg/g FAE)	Flavonoid compounds	Concentration (μg/g FAE)
Chlorogenic acid	4.22	Naringin	12.45
Syringic acid	13.12	Rutin	9.02
Cinnamic acid	7.00	Quercetin	8.22
Pyrogallol	9.42	Kaempferol	7.36
Gallic acid	2.06	Apigenin	15.22
Ferulic acid	8.89		

## Discussion

Due to the recent growth in food demand, agricultural expansion, and increased crop productivity have resulted in a rise in the use of many synthetic products in the fields, which has created a number of issues for the environment, farmers, and consumers^78^. As a result, the use of plant biostimulants is a novel, ecologically friendly strategy for sustainable crop production, which is constrained by issues including water shortages, resource depletion, environmental stresses, and climate change^33^.

Coriander plants must be provided with nitrogen during the growing season in order to accomplish the necessary increase in vegetative growth and quality improvement and production^79,80^. Moreover, N fertilization affects the accumulation of phytochemicals in the plant, including phenolic compounds, and nitrogen deficiency, which may lead to an increase in their concentration in the plant^81^.

It is ideal to comprehend the balance between using the proper amount of nitrogen for plant growth and production, especially in leafy green vegetables like the coriander that was the subject of this study. With less of the suggested mineral N fertilizer for coriander growing, this study assessed the biostimulant effect of *Acacia saligna* aqueous fruit extract at various concentrations. The overuse of N fertilizers and their improper application are two major issues that restrict the yield of leafy crops. Leafy vegetables can have toxicity issues as a result of excessive fertilization^9,82^. As a result, it is advised to use natural plant biostimulants as a promising and creative strategy to ensure better and long-lasting yields while minimizing the usage of inorganic fertilizers^83^.

The spraying of various *A. saligna* extract concentrations had an impact on vegetative development and yield in this consistency. The findings usually showed that the application of a concentration of *A. saligna* FAE (4%) combined with 50% N fertilizer, improved coriander plant production and vegetative development. The abundance phytochemicals found in the extract, including phenolic and flavonoid components, may be responsible for these results. According to the findings of earlier research, the various botanical parts of *A. saligna* contain a variety of powerful compounds that promote the growth of many plants, including proteins, carbohydrates, phenolic, gallic acid, p-coumaric acid, palmitic, oleic, and linoleic acids^60,84^. By reducing the negative impacts of stress, phenolic compounds demonstrated a biostimulant action on many plant species. Most likely, phenolic chemicals help to combat various stressful situations by triggering the body's antioxidant defense mechanism. Terpenes and phytohormones also have a favorable impact through seed priming and foliar spraying^37^. Furthermore, certain extracts, such as those from the *Moringa oleifera* leaf, which is rich in proteins, lipids, carbohydrates, minerals, vitamins, and amino acids, have a simulative effect on plant growth and productivity on the okra and tomato plants^85–87^. Utilizing plant biostimulant increased the overall yield of the leaves produced by the spinach and lettuce plants^88^.

Chlorophyll a, chlorophyll b, and chlorophylls a + b contents were reached the highest amount in plants treated with *A. saligna* FAE 4% + 50% N fertilizer while carotenoids content showed the highest amount in plants treated with *A. saligna* FAE 4% + 50% N fertilizer and *A. saligna* FAE 4% + 50% N fertilizer. A significant number of secondary metabolites are phytotoxic and have an allelopathic effect in addition to their biostimulant activity on plant development and seed germination^89,90^. Fruit extract from *A. saligna* might be regarded as a biostimulant to enhance vegetative growth and production because of the chemicals found in it, which called allelochemicals^55,91^.

The methanolic extracts of *A. saligna* had a greater impact on the seedling length of the plant diversity along Egypt's Nile Delta Coast than the aqueous extracts, but only at low doses^54^. Two agricultural crops, wheat and canola, showed persistent declines in their vegetative growth parameters (shoot and root length, fresh and dry weight), as the allelopathic extract concentration from leaves and stems of *A. saligna* increased to 10%^56^. *A. saligna* leaf and root aqueous extract concentrations (0%, 5%, 10%, 15%, and 20%) significantly increased the inhibition of seed germination and the lengthening of shoot and root, which gradually reduced the seedling performance of three native Mediterranean shrubs (*Astragalus armatus* Willd., *Retama raetam* (Forssk.) Webb & Berthel., and *Helianthemum kahiricum* Del.)^92^. According to another study, stem and leaf extracts of *A. saligna* contain significant amounts of allelochemicals that hinder the growth and development of wheat, radish, barley, and arugula. This reduces crop biomass and has an negative impact on other related parameters at concentrations of 5–25%^55^.

Researchers and consumers are paying increasing attention these days to fruits and vegetables high in phytonutrients. Phytoalexins, antioxidants, and signaling molecules are the roles played by these substances, which are secondary metabolites produced by plants from primary metabolites^40,93^. They also aid in the defense mechanisms used by plants to respond to stress. By altering plant biochemical, molecular, and physiological processes, plant-derived biostimulants are able to enhance plant growth, water usage efficiency, nutrient uptake, tolerance to abiotic and biotic challenges, and photosynthesis^26,40,94,95^.

Coriander leaves are regarded as a significant source of biological substances. In the present investigation, different concentrations of *A. saligna* FAE had a significant impact on biochemical compounds (antioxidant, flavonoid, total phenolic, and carbohydrates) and total soluble content. In this sense, using biostimulants raised the biochemical components of a variety of leafy vegetables while improving their quality^39,96,97^.

Low essential oil (EO) percentages from the leaves, in the range of 00.06–0.13%, were observed. The least amount of EOs was observed in *Coriandrum sativum* (0.12%) among the EOs extracted by hydrodistillation from several medicinal plants^98^. Numerous chemicals, including (*E*)-2-decenal, 1-methoxymethoxy-oct-2-yne, (*E*)-2-decen-1-ol, 3,5,5-trimethyl-1-hexene and phytol were present in treated and untreated plants' EOs. The main constituent in the EO from the leaves was (E)-2-decenal, which followed by decanal, dodecanal, (*E*)-2-tridecenal and (*E*)-2-dodecenal^47^. Decanal, trans-2-decenal, 2-decen-1-ol, cyclodecane, *cis*-2-dodecenal, dodecan-1-ol and dodecanal were identified as the major components in the leaf EOs^73^. In the leaf EO, (*E*)-2-decenal made up the majority of aldehydes (52.2%), whereas 1-decanol made up the majority of monoterpene alcohols^77,99^.

The primary constituents decanal, cis-phytol, 1-tetradecanol, (E)-2-dodecenal, dode-canal, n-decanol, and trans-2-undecen were present in both fresh and dried coriander plants in the isolated coriander EOs^74^. The main chemicals found in coriander leaves EO were (*E*)-2-decenal, linalool, (*E*)-2-dodecenal, (*E*)-2-tetradecenal, 2-decen-1-ol, (*E*)-2-undecenal, dodecanal, (*E*)-2-tridecenal, (*E*)-2-hexadecenal, pentadecenal, and *α*-pinene^66^. Another study showed that the coriander plant EO contained the largest concentration of aliphatic aldehydes (decanal, *E*-2-dodecanol and *E*-2-decenol), as well as phytol, linalool, and oleic acid^76^. n-Decanal, 2*E*-dodecanal, 2*E*-decanal, 2*E*-tridecen-1-al, and n-nonane made up the majority of the leaf EO^100^. Additionally, the EO from coriander stems revealed that phytol (61.86%) was the primary component present^101^. EOs from coriander leaves that were extracted using two different procedures and had linalool as the primary component (51.32% and 61.78%, respectively) were analyzed by GC-MS^102^.

The presence of various phenolic compounds, including 7-hydroxyflavone, naringin, rutin, quercetin, kaempferol, luteolin, apigenin, and catechin, in varying amounts, was seen in coriander plants treated with the four treatments. These substances play a significant role in the antibacterial and antioxidant actions^103–106^.

Aqueous coriander extract was shown to include phenolic components such catechol, salicylic acid, glycitin, pyrogallol, gentisic acid, protocatechinic acid, quinic acid, and caffeic acid^107^. Aerial parts of *C. sativum* were found to contain the phenolic chemicals apigenin, catechin, and p-coumaric acid, as well as aliphatic alkenals and alkanals^108,109^.

Different plant parts, their various developmental stages, and environmental alterations all have an impact on the composition of phytochemicals^110^. These elements affect the biosynthetic pathways of the plant, which in turn affects the relative proportion of the primary constituents. Numerous studies have demonstrated that biostimulants affected the production and content of EOs^111–113^. Plants create phytochemicals for defense and communication through secondary metabolic pathways.

## Conclusion

Under field conditions, coriander's phytochemicals and growth productivity responded to the application of *Acacia saligna* extract biostimulant (2, 4, and 6%) combined with 50% N fertilizer, were evaluated. The treatment of *A. saligna* plant extract at 4% + 50% N fertilizer was found to have positive impacts on coriander plants' vegetative development, green yield, and phytochemicals. The findings showed that coriander plants generally produced more leaves per plant, had heavier plants overall (g), and produced a greater overall yield (kg/m^2^). The use of plant extracts as a biostimulant may assist reduce environmental pollution caused by the use of mineral nitrogen fertilizers by reducing the amount used in agriculture, which may leak into the ecosystem.

### Supplementary Information

Supplementary Information.

## Data Availability

All data generated or analyzed during this study are included in this published article.
