# Supplementary material for: Growth, productivity and phytochemicals of Coriander in responses to foliar application of Acacia saligna fruit extract as a biostimulant under field conditions
Source: Sci Rep. 2024 Feb 5;14:2921. doi: 10.1038/s41598-024-53378-5 (PMC10844193; doi:10.1038/s41598-024-53378-5)
Supplement: Supplementary file 1 — Supplementary Information. [file 41598_2024_53378_MOESM1_ESM.docx]

Growth, productivity and phytochemicals of Coriander in responses to foliar application of *Acacia saligna* fruit extract as a biostimulant under field conditions

Alkharpotly, A. A. ^1,2^, Doaa Y. Abd-Elkader ^3^, Mohamed Z. M. Salem ^4,^*, and Hanaa S. Hassan ^3^

^1^ Horticulture Department, Faculty of Agriculture and Natural Resources, Aswan University, Aswan, Egypt. [alkharpotly@agr.aswu.edu.eg](mailto:alkharpotly@agr.aswu.edu.eg)

^2^ Horticulture Department, Faculty of Desert and Environmental Agriculture, Matrouh University, Marsa Matrouh, Egypt.

^3^ Department of Vegetable, Faculty of Agriculture (EL-Shatby), Alexandria University, Alexandria 21545, Egypt; [doaa.abdelkader@alexu.edu.eg](mailto:doaa.abdelkader@alexu.edu.eg) (D.Y.A.-E.); [hanaa.saad@alexu.edu.eg](mailto:hanaa.saad@alexu.edu.eg) (H.S.H.)

^4^ Forestry and Wood Technology Department, Faculty of Agriculture (El-Shatby), Alexandria University, Alexandria 21545, Egypt; [mohamed-salem@alexu.edu.eg](mailto:mohamed-salem@alexu.edu.eg)

**Corresponding author: mohamed-salem@alexu.edu.eg**

| 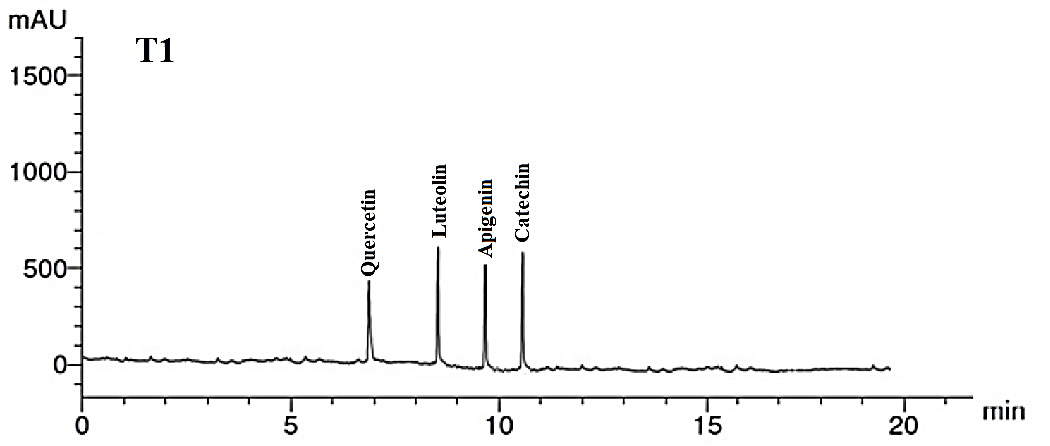 |
| --- |
| 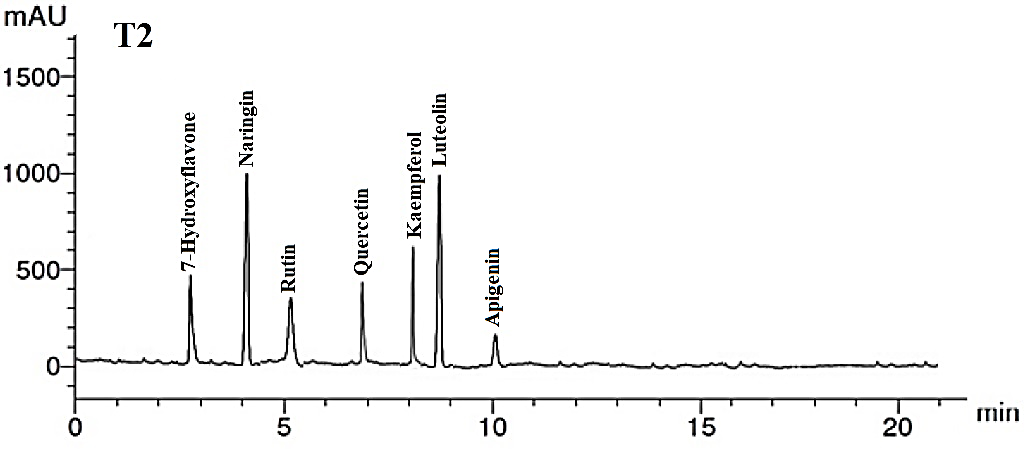 |
| 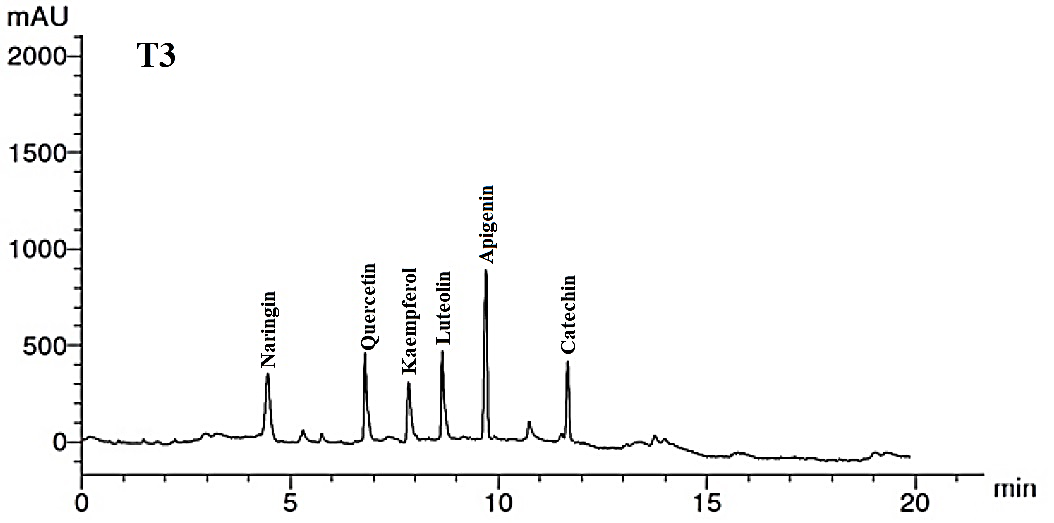 |
| 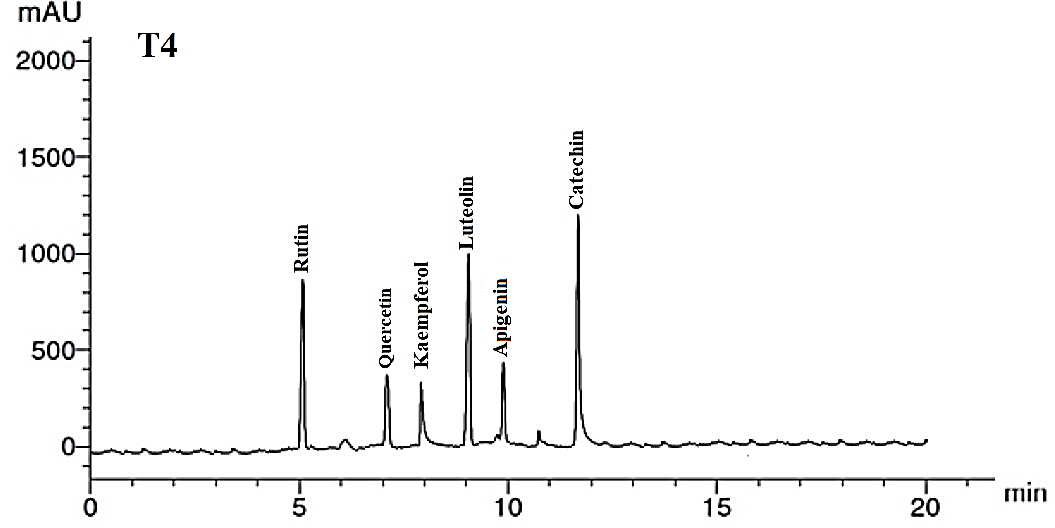 |

**Figure S1.** The identified flavonoid compounds from the methanol extract of *C. sativum* leaves by HPLC analysis. T1: Control; T2: *A. saligna* FAE 2% *+* 50% N fertilizer; T3: *A. saligna* FAE 4% *+* 50% N fertilizer; T4: *A. saligna* FAE 6% *+* 50% N fertilizer.


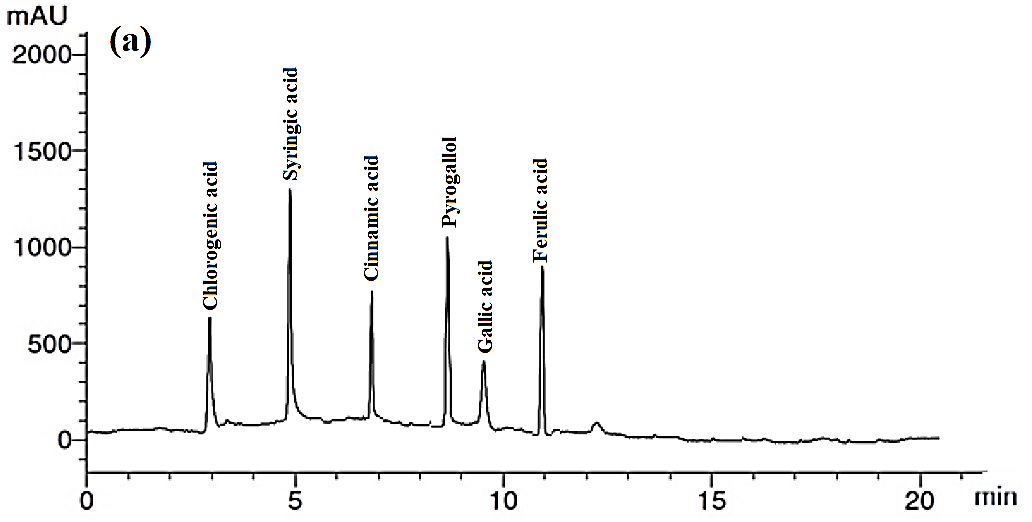


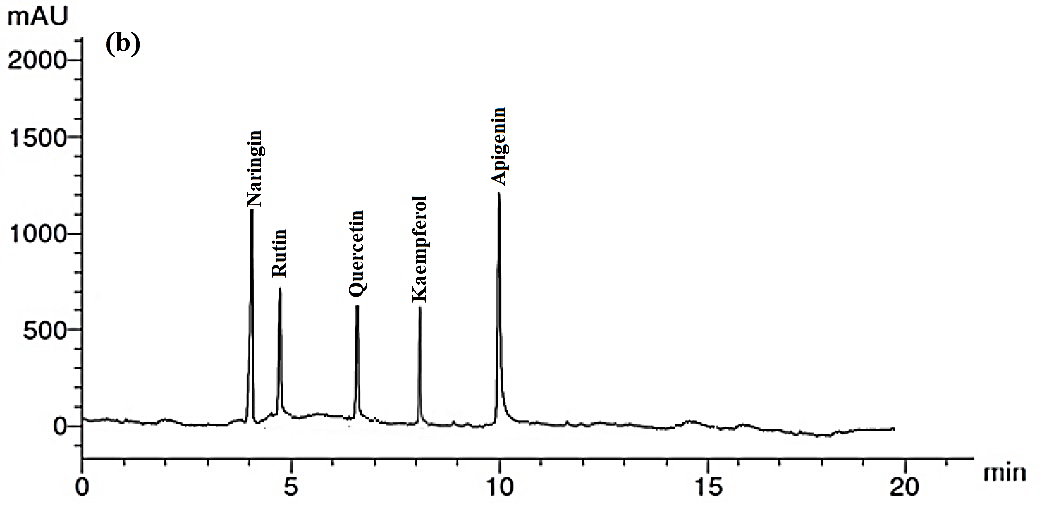


**Figure S2.** The identified phenolic (a) and flavonoid (b) compounds in *Acacia saligna* FAE by HPLC analysis
